# circSKA3 promotes colorectal cancer metastases through miR-1238 and methylation

**DOI:** 10.1007/s11010-023-04773-5

**Published:** 2023-05-31

**Authors:** Yonghuan Mao, Ji Miao, Ling Xi, Hanwen Tong, Xiaofei Shen, Qiang Li, Chunzhao Yu

**Affiliations:** 1 Department of General Surgery, Nanjing Drum Tower Hospital Clinical College of Nanjing Medical University, Nanjing, 210008 China; 2 Department of General Surgery, the Second Affiliated Hospital of Nanjing Medical University, Nanjing, 210011 China; 3 Department of Gerontology, Jiangsu Province Official Hospital, Nanjing, 210009 China; 4 Department of Emergency, Nanjing Drum Tower Hospital Clinical College of Nanjing Medical University, Nanjing, 210008 China; 5 Department of General Surgery, Sir Run Run Hospital of Nanjing Medical University, Nanjing, 211112 China

**Keywords:** circSKA3, CRC, miR-1238, Methylation

## Abstract

Colorectal cancer (CRC) is becoming one of the most common cancers overworld, which causes a high rate of death in patients. circRNAs are non-coding RNAs(ncRNAs), which have been reported to be involved in the development of many cancers, including CRC. However, the exact mechanism that how circRNAs function through in CRC remains unclear. In this study, we firstly used GEO database and bioinformatic methods to identify the significant changed circRNAs, with circSKA3 being the most significantly upregulated circRNAs in CRC tissues. PCR results further confirmed higher expression of circSKA3 in CRC patients. CCK-8, scratch, and transwell assays indicated that circSKA3 could promote the proliferation, migration, and invasion of CRC cell lines for cell detection. Dual-luciferase assays were carried out to detect the downstream targets of circSKA3, and a binding site between circSKA3 and miR-1238 was identified and miR-1238 could also combine with YTHDF2. Overexpression of YTHDF2 rescued the decreased cell proliferation, migration, and invasion caused by miR-1238 overexpression. RIP assay further indicated that YTHDF2 could decrease the methylation of STAT5A. In summary, our study found that circSKA3 was upregulated in CRC tissues comparing with normal tissues. circSKA3 could increase the expression ofYTHDF2 through sponging miR-1238 to decrease the methylation of STAT5A, which could provide a novel target for CRC treatment.

## Introduction

Circular RNAs (circRNAs) have been discovered more than 40 years ago [1], and are defined as a closed-loop through back-splicing through RNA polymerase II to link the 3′-end splice sites with the upstream 5′-end splice site [2].With the development of sequencing technology, more and more circRNAs have been identified and proven to be of great significance in biological processes. However, lots of circRNAs are recognized as rubbish during the transcription. circRNAs play important roles in multiple systems, such as nervous system [3], endocrine system [4], circulatory system [5], and respiratory system [6]. They function through miRNAs sponging [7], cRBPs interaction [8], protein translation [5], and transcriptional or post-transcriptional gene expression regulation.

Colorectal cancer (CRC) is one of the most common cancers in the world, which could cause almost 600,000 deaths every year [9, 10]. The most recognized options for CRC patients treatment include surgery and chemotherapy [11]. With progresses in strategies to treat CRC, the prognosis of CRC patients has been improved tremendously. Although great advances in the CRC diagnosis and treatment have been achieved, the gene expression disorder and exact molecular mechanisms have not been fully addressed in CRC. Emerging evidence has shown that circRNAs also play a crucial role in CRC development and progression. circular RNA circ_0068464 was identified as a novel target, which could interact with microRNA-383 to regulate Wnt/β-catenin pathway in colorectal cancer [12]. circPTEN1, a circular RNA generated precursor mRNA PTEN, could generate circPTEN1 and inhibit TGF-β/Smad pathway to suppress cancer progression c [13]. Moreover, Hsa_circ_0062682 also promotes Colorectal Cancer tumor growth by sponging miR-940 to regulate expression of PHGDH to improve serine metabolism [14]. These researches indicate the importance of circRNAs in CRC development and progression.

Our study uncovered that circSKA3 was upregulated in CRC. CircSKA3 could increase the expression of YTHDF2 through sponging miR-1238 to decrease the methylation of STAT5A, which could provide a novel target for CRC treatment.

## Materials and methods

### Clinical samples

CRC cancer tissue samples and para-carcinoma tissue samples were taken from 20 CRC patients according to surgical procedure. All the tissue of the patients were asked and provided written consent. This study was involved in human participants and approved by Ethics Committee.

### Cell culture and transfection

CRC cell lines SW620 and HCT116 were cultured in DMEM (GIBCO, USA) combined with 10% fetal bovine serum (Cromwell, USA) and supplemented with penicillin and streptomycin (Sigma-Aldrich, USA). The cell lines were cultured at 37 °C with 5% O_2_. Overexpression plasmid pcDNA-circSKA3 and small interfering RNA of circSKA3 (si- circSKA3) was transfected into cells by using Lipo3000 (Invitrogen, Carlsbad, CA, USA). The plasmid or miR-1238 mimics were constructed and purchased from by Genepharm (Shanghai, China).

### RNA isolation and qRT-PCR

RNA isolated from cells and reversed according to manufacturer’s instructions. The RNA was amplified according to manufacturer’s instructions. The gene expression was calculated using 2^–ΔΔCt^ method. Primer list: circSKA3 (F: 5′-CCCACCTACCAAACAATCACT-3′, R: 5′- GGCTTCTTGCTCGTGTACCT -3′), GADPH (F: 5′-AACGGATTTGGTCGTATTG-3′, R: 5′-GGAAGATGGTGATGGGATT-3′).

### CCK8 assay

Transfected cell lines were cultured and seeded in 96-well plates at a density of 2,000. A CCK8 assay kit (Sigma, USA) was performed to checked living cells according to manufacturer’s instructions. The absorbance of buffer was detected by a microplate reader with five times. All measurements were carried out at three times.

### Wound-healing assay

Cell lines (5 × 10^5^) were planted in a 6-well plate, A was used to scratch to generate A linear gap was generated by a 200 μl pipette tip. After 24 h, the same place was got pictures by the microscope and IPP software was used to measure the width (W) of the scratch wound. All measurements were carried out at three times.

### Transwell assay

After treatment, cell was digested and suspended. Then, added into transwell chamber inserts (Millipore, USA), added with matrige. 24 h later, the cells at the bottom were stained and pictures taken by microscope. Then, the cell was count by Image J in accordance with the manufacturer's instructions.

### Luciferase assay

The wildtype of circSKA3, 3′-UTR of YTHDF2 and their mutated (MUT) plasmids were constructed by GENEWIZ (Shanghai, China). MiR-NC or miR-1238 mimics was added into luciferase reporter vectors and transfected into cells. The cells were lysed after 24 h post-transfection to check the luciferase activity.

### RIP assay

Magna RIP RNA Binding Protein Immunoprecipitation Kit (Bersinbio, Guangzhou, China) was used for the RIP assay according to the manufacturer’s protocol. Cells were lysed in RIP lysis buffer and incubated with anti-YTHDF2 antibody (Millipore, Billerica, MA, USA) or non-specific (Millipore) with rotation at 4 °C overnight. Anti-IgG antibody was used as a negative control. Then, magnetic beads were added and incubated at 4 °C for 1 h. After Proteinase K treatment, the enriched RNA amplified by qRT-PCR.

### Statistical analysis

SPSS IBM 22.0 was used for statistical analysis. The P-values were determined by using student’s t test t or Analysis of Variance (ANOVA). All data in the graphs are showed as mean ± SD.

## Results

### Upregulation of circSKA3 in CRC tissues

**Fig. 1:**
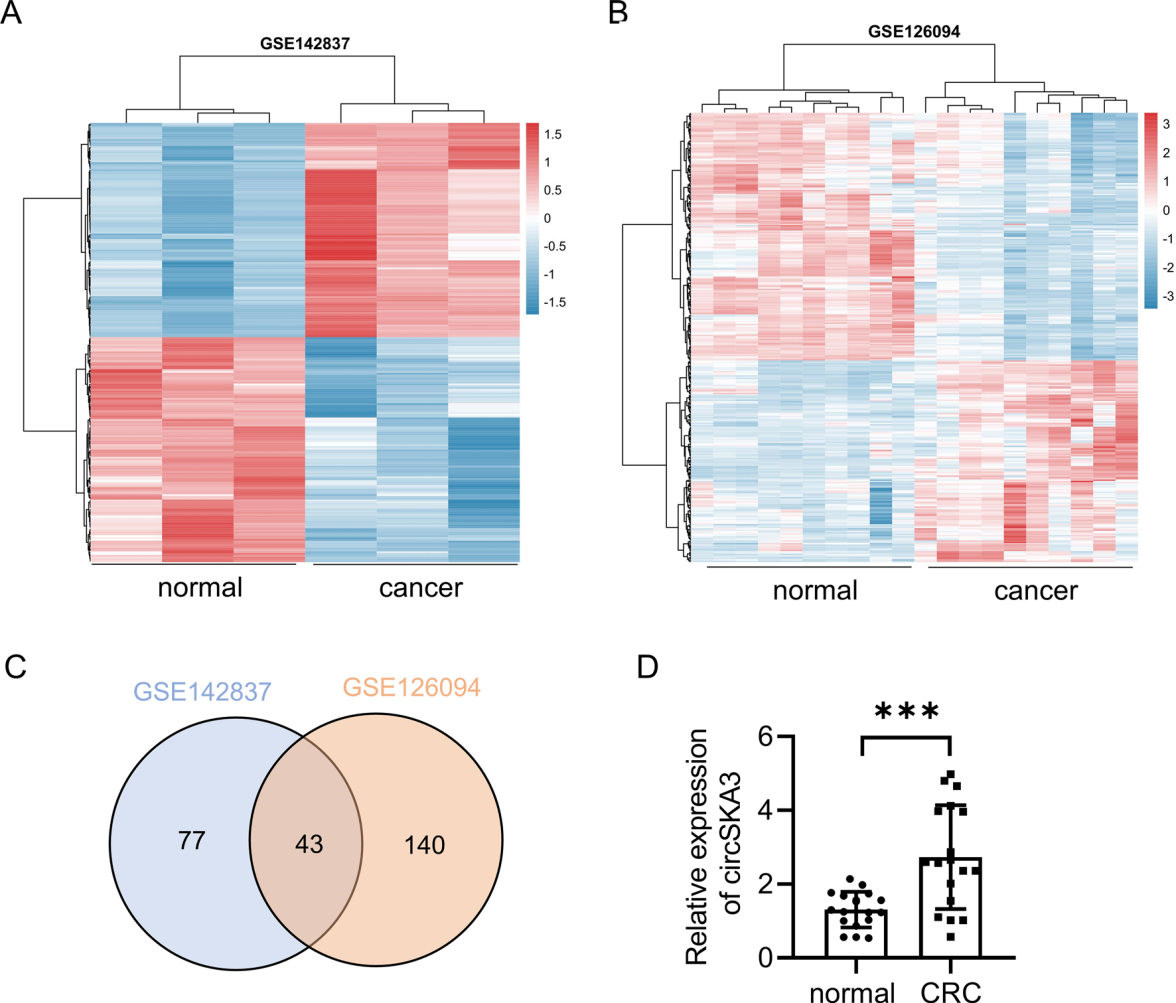
circSKA3 was overexpressed in CRC tissues. **A**–**B** Heat maps of differentially expressed circRNAs in CRC tissues and non-tumor tissues obtained from GSE142837 and GSE126094. **C** The numbers of overlapping differentially expressed circRNAs from two GEO databases were shown in venn diagram. **D** The level of circSKA3 expression was evaluated by qRT-PCR in 20 paired CRC tissues and non-tumor tissues. **p < 0.01

To figure out differentially expressed circRNAs in CRC, we used different GEO databases to screen circRNAs that may be differentially expressed in CRC tissues and non-tumor paired tissues from the GEO databases (GSE142837 and GSE126094) (Fig. 1A, B). A total of 43 upregulated circRNAs were identified in two GEO databases (Fig. 1C).CircSKA3 is one of highest expression of circRNAs in the databases. So, we chose it for further study. In 20 samples of CRC tissues from CRC patients, circSKA3 was highly expressed in CRC cancer tissues, which was compared with para-carcinoma tissues (Fig. 1D). Collectively, the above data indicated that circSKA3 might be involved in CRC progression.

### circSKA3 promoted the malignancy of CRC cells

**Fig. 2:**
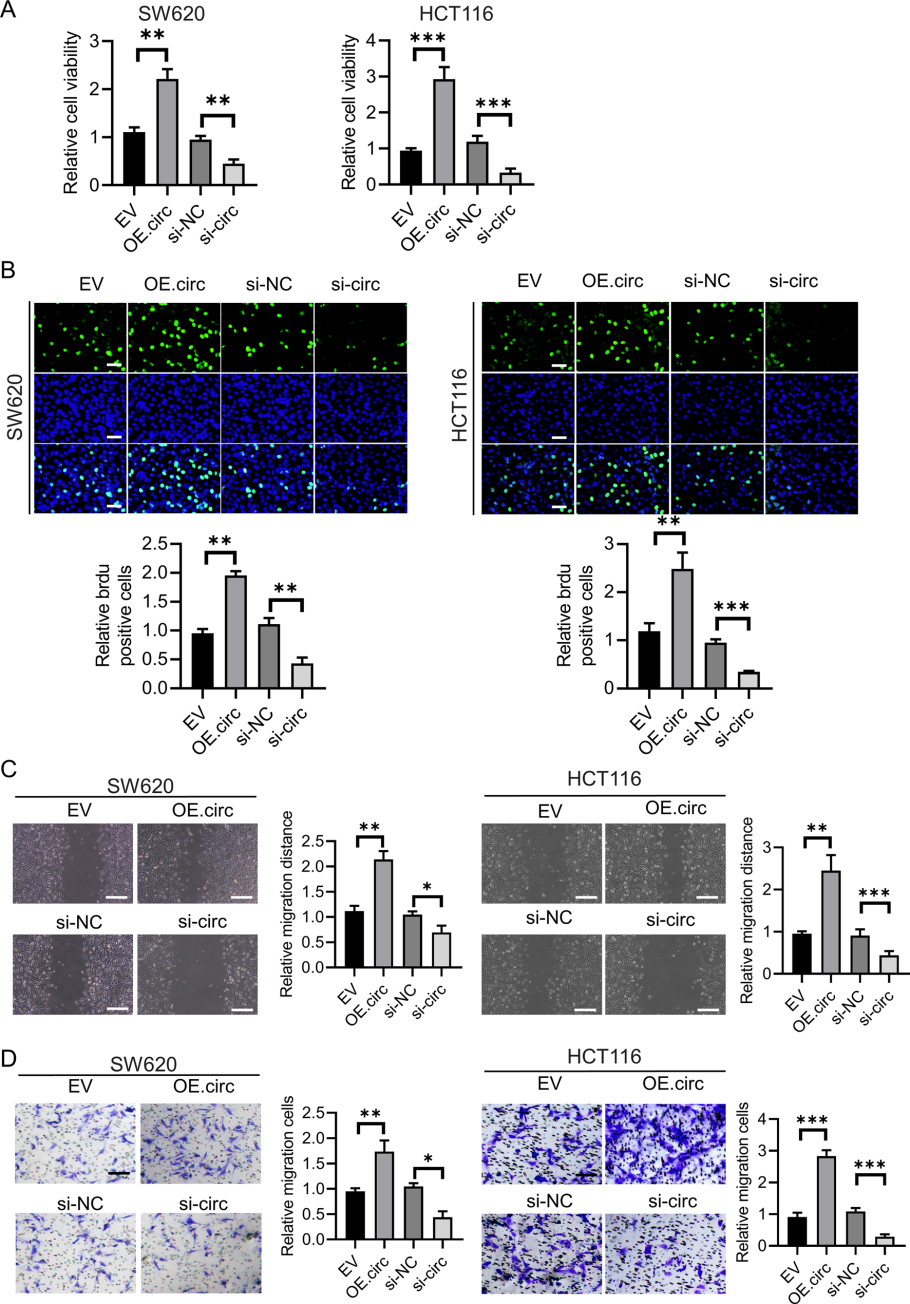
circSKA3 functions as an oncogene in CRC cells. **A** Effect of si- circSKA3 and OE- circSKA3 on proliferation in CRC cell lines by a CCK8 assay. **B** EDU staining was carried to detect the cell proliferation. **C** A wound-healing assay was carried out in CRC cells treated with si- circSKA31 and OE- circSKA3 (scale bars, 200 μm). **D** A transwell assay was carried out in CRC cells treated with si- circSKA3 and OE-circSKA3 (scale bars, 100 μm). *p < 0.05, **p < 0.01, ***p <0.001

In order to confirm and identify the function of circSKA3 in CRC progression, we constructed overexpression plasmid pcDNA-circSKA3 (OE.circ) and small interfering RNA of circSKA3 (si-circ). CCK-8 assay was carried out to detect the viability after si-circSKA3 and pcDNA-circSKA3 transfection, which was showed in Fig. 2A. The results showed that circSKA3 overexpression significantly enhanced growth rate of cell lines and circSKA3 knockdown inhibited the cell viability. And Edu staining showed similar result (Fig. 2B). Then, the wound-healing assay suggested that overexpression of circSKA3 increased the wound healing area, while si-circSKA3 exhibited a lower migration ability (Fig. 2C). Transwell assay showed the same results (Fig. 2D). These results indicated that circSKA3 could promote CRC malignancy.

### circSKA3 suppressed miR-1238 expression

**Fig. 3:**
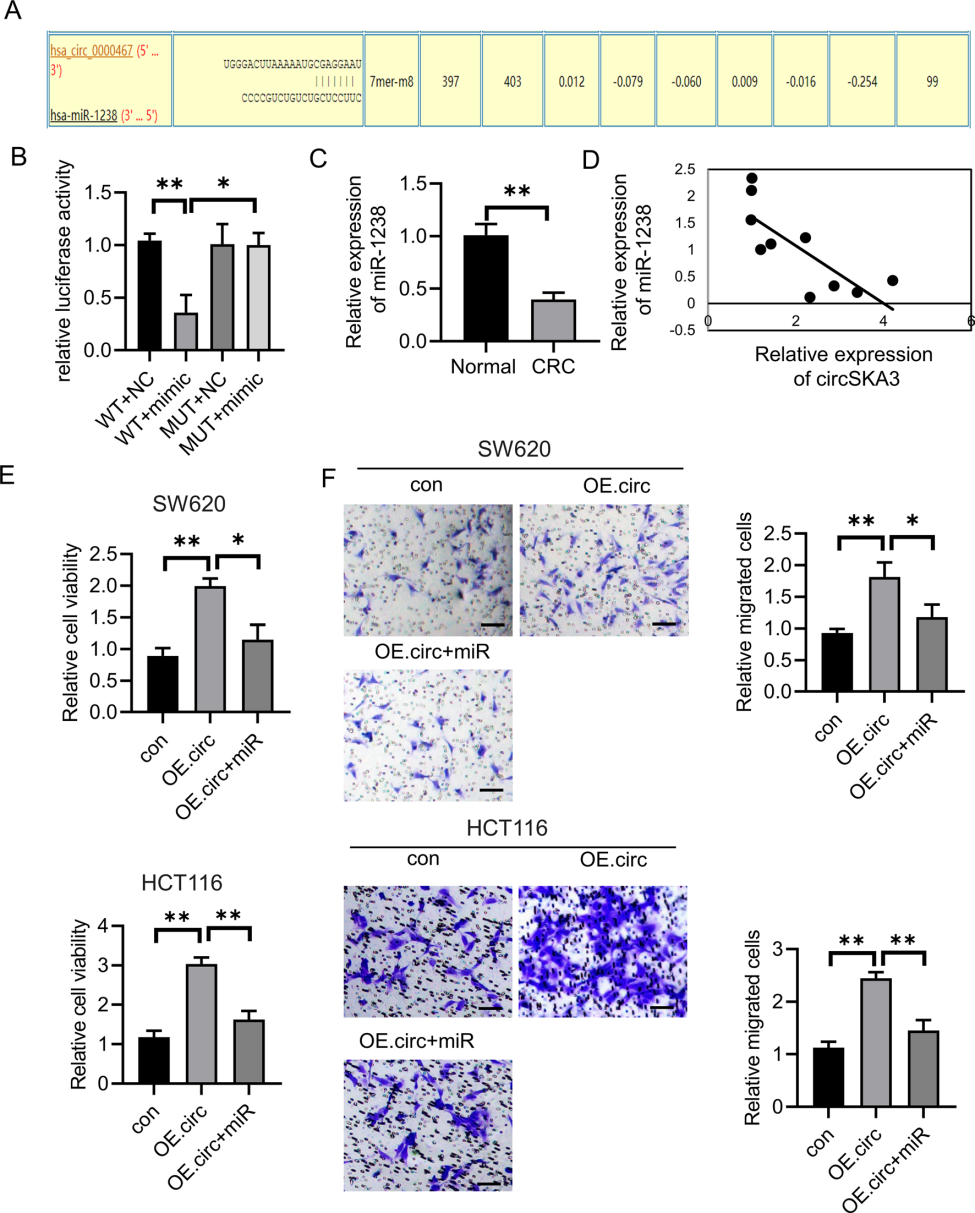
CRC could interact with miR-1238. **A**–**B** Predicted miR-1238 binding sites in circSKA3 and dual luciferase report assay in WT-circSKA3 or MUT-circSKA3 co-transfected with miR-NC or miR-1238 mimics. **C** miR-1238 expression in 20 paired CRC tissues and non-tumor tissues. **D** Pearson's correlation analysis was used to check the relationship between circSKA3 and miR-1238. **E**–**F** Cell proliferation and migration after transfected with miR-1238 and oe circSKA3. *p < 0.05, **p < 0.01

One of mechanisms for circRNAs to regulate the gene expression is sponging microRNAs. In order to further clarify the exact mechanism of circSKA3 in CRC, we used bioinformatic databases to identify microRNAs, which possesses biding sites with circSKA3. It indicated that miR-1238 showed binding sites with circSKA3 and gained a high score (Fig. 3A). Luciferase assay showed that wild type (WT)- circSKA3 inhibited luciferase activity, but not mutant (MUT)- circSKA3 (Fig. 3B). Additionally, the level of miR-1238 was decreased in CRC tissues (Fig. 3C). Pearsons analysis indicated that the expression of miR-1238 was negative with circSKA3 (Fig. 3D). Then, we overexpressed the miR-1238 upon circSKA3 overexpression. The results showed that miR-1238 overexpression reduced the cell viability and migration caused by circSKA3 overexpression (Fig. 3E–F).

### MiR-1238 directly targeted YTHDF2

**Fig. 4:**
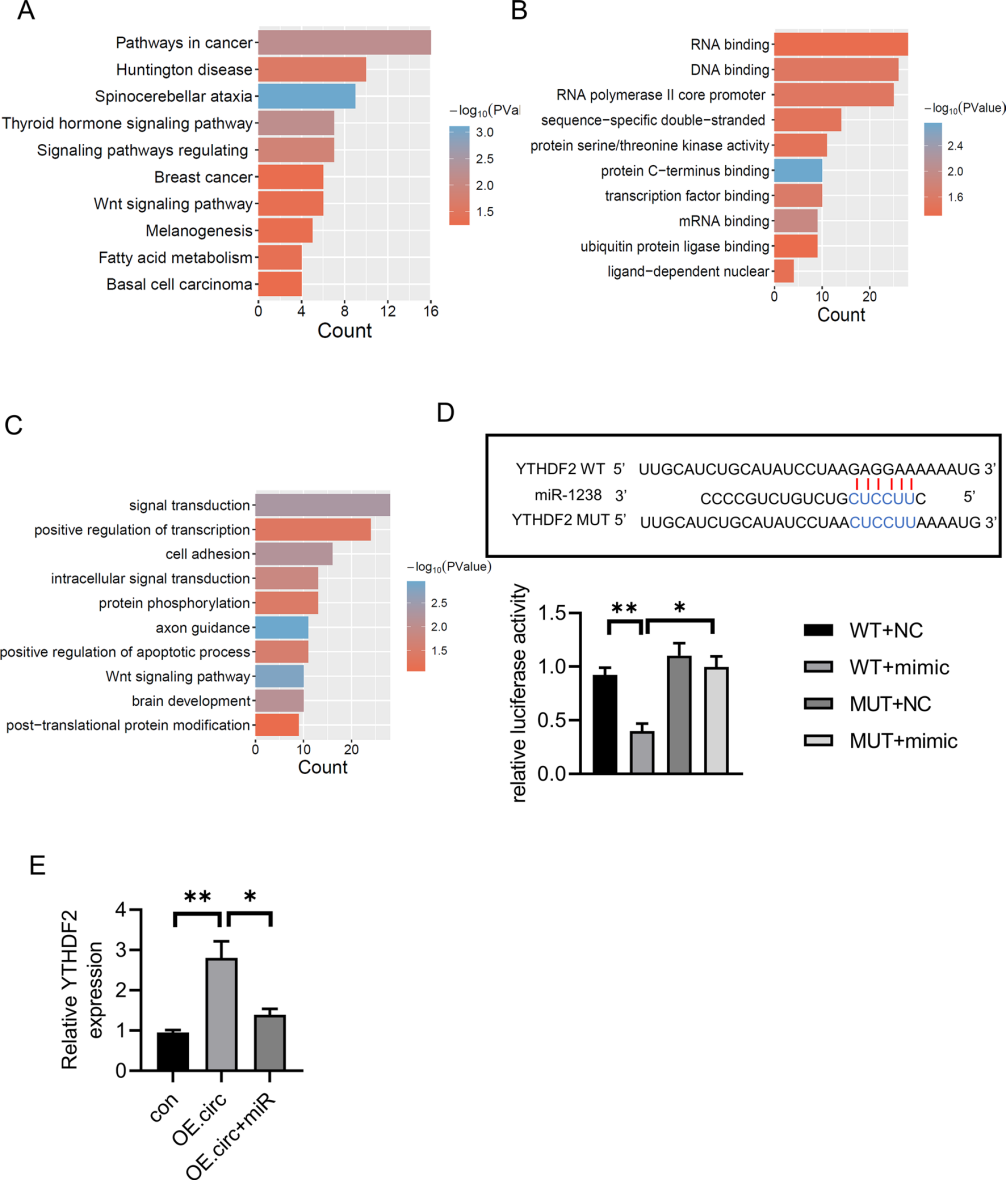
miR-1238 interacts with YTHDF2. **A** KEGG analysis of miR-1238 targeted genes. **B–C** molecular function and biological process of miR-1238 targeted genes. **D** Predicted miR-1238 binding sites in 3’UTR of YTHDF2 and dual luciferase report assay in WT- YTHDF2 or MUT- YTHDF2 co-transfected with miR-NC or miR-1238 mimics. *p < 0.05, **p < 0.01

MiRNAs could combine with 3’UTR of target gene. We used the bio-informatic tools to identify the miR-1238 targeted genes. And KEGG pathway analysis indicated that the targeted genes were enriched in pathways in cancer (Fig. 4A). GO and biological process implied that the target gene may take part in RNA binding and regulation of apoptotic process (Fig. 4B–C). According to systematic analysis, YTHDF2 showed target sites and gained a high score (Fig. 4D). The luciferase activity of WT- YTHDF2, but not mutant YTHDF2, was decreased in the miR-1238 mimic group compared with NC group (Fig. 4D). Furthermore, circSKA3 overexpression increased the expression of YTHDF2, while miR-1238 significantly inhibited its expression in CRC cells (Fig. 4E).

### circSKA3 promoted CRC growth via YTHDF2/STAT5A axis

**Fig. 5:**
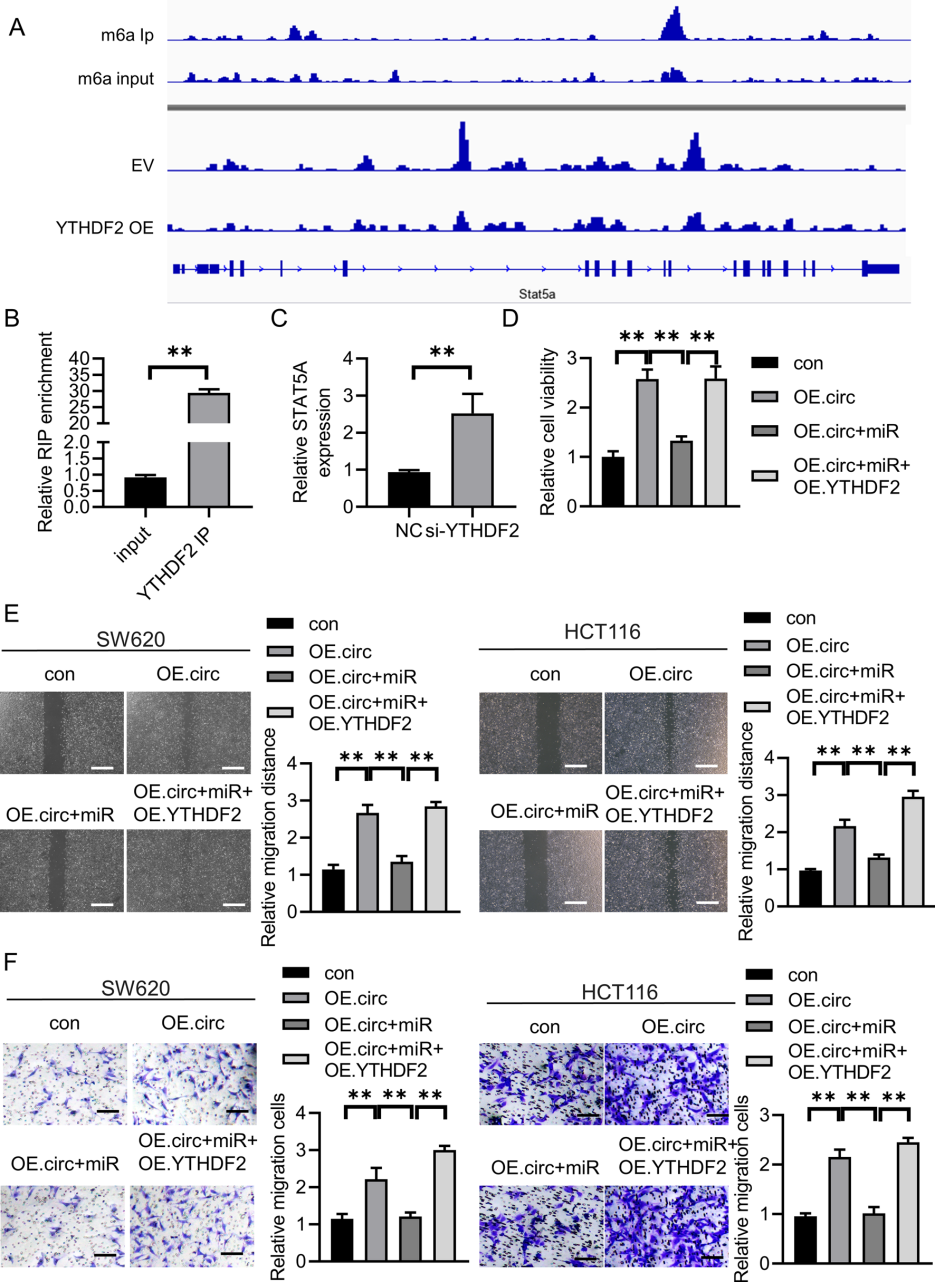
YTHDF2 overexpression rescues the decreased proliferation, migration, and invasion by miR-1238 overexpression. **A **STAT5A methylation was analyzed by bioinformatic database. **B** RIP-RT-PCR was used to confirm that the YTHDF2 could combine with STAT5A mRNA. **C** STAT5A expression was detected by YTHDF2 knockdown. **D**–**F** YTHDF2 overexpression rescues the decreased proliferation, migration, and invasion by miR-1238 overexpression according to CCK8 assay, scratch assay, and transwell assay. **p < 0.01

YTHDF2 is an m6A reader that could recognize m6A mRNA to mediate m6A transcripts degradation. YTHDF2 was identified to promote multiple myeloma cell proliferation via STAT5A/MAP2K2/p-ERK axis [15]. First, we used other GEO database (GSE162004, GSE120860) to improve that STAT5A methylation in gastrointestinal tumors (Fig. 5A). We applied YTHDF2-RIP RT-qPCR to evaluate the relationship between YTHDF2 and STAT5A. It indicated that YTHDF2 could combine with STAT5A mRNA in CRC cells (Fig. 5B). And knockdown of YTHDF2 could improve the expression of STAT5A(Fig. 5C).We overexpressed circSKA3 in CRC cells with miR-1238 overexpression and YTHDF2 overexpression. circSKA3 accelerated cell viability, migration, and invasion in CRC cells (Fig. 5D–F). Moreover, overexpression of miR-1238 reversed the promoting effect of CRC cells by circSKA3 overexpression (Fig. 5A–C). Finally, YTHDF2 overexpression enhanced the cell migration and viability reduced by miR-1238 overexpression.

## Discussion

More and more evidence has indicated that circRNAs play crucial roles in human cancers including CRC. For example, CircIL4R could sponge miR-761 to regulate TRIM29/PHLPP1 axis to activate the PI3K/AKT signaling pathway to promote proliferation and invasion in CRC cancer [16]. Exosome could transmit circCOG2 to induce colorectal cancer metastasis through miR-1305/TGF-β2/SMAD3 axis [17]. Moreover, circMYH9 could also promote colorectal cancer growth in a p53-dependent manner by regulating serine metabolism and redox homeostasis [18].

One of exact mechanisms for circRNAs to regulate gene expression is to act as ceRNAs to reduce the miRNA expression. For example, ciRS-7 could sponge miR-7 to function as an oncogene and induce the tumor progression in many types of cancers [19]. Circ_0062682 was localized in the cytoplasm predominantly and act as a ceRNA to sponge miR-940 to promote the tumor progress [14]. In this study, circSKA3 was upregulated in CRC tissues. circSKA3 could function as an oncogene to promote cell proliferation, migration, and invasion. We used online databases to predict and verified that miR-1238 could combine with circSKA3. It indicated that circSKA3 acted as a ceRNA to sponge the miR-1238 and decreased the expression of miR-1238. Therefore, circSKA3 was a negative regulator of miR-1238 expression. In molecular function validation, miR-1238 overexpression reduced the cell viability and migration caused by circSKA3 overexpression.

YTHDF2 is an m6A reader that could recognize m6A mRNA to mediate m6A transcripts degradation [20]. YTHDF2 was identified to promote multiple melanoma cell proliferation via STAT5A/MAP2K2/p-ERK axis [15]. YTHDF2 was reported to act as an oncogene in lung cancer [21]. However, YTHDF2 functions as a revisable role in hepatocellular carcinoma to disrupt ERGF mRNA stability [22]. Previous research has reported that YTHDF2 plays crucial roles in CRC [23]. We identified that YTHDF2 was overexpressed in the CRC and YTHDF2 overexpression enhanced the cell migration and viability reduced by miR-1238 overexpression. For the downstream of YTHDF2, we used GEO databases to identify the STAT5A in gastrointestinal tumors. And RIP-PCR confirmed that YTHDF2 could combine with STAT5A.

In conclusion, we revealed that circSKA3 could promote the development of CRC by enhancing the expression of YTHDF2 via sponging miR-1238. Our study might provide a novel target for treatment of CRC.

## Data Availability

Enquiries about data availability should be directed to the authors.
